# Recording blood pressure and eGFR in primary care after the Belgrade screening study

**DOI:** 10.1080/0886022X.2018.1450759

**Published:** 2018-03-22

**Authors:** Visnja Lezaic, Jelena Marinkovic, Zoran Milutinovic, Nada Jovanovic-Vasiljevic, Vesna Vujicic, Branka Pejovic, Snezana Kalabic, Ljubica Djukanovic

**Affiliations:** a School of Medicine, University of Belgrade, Belgrade, Serbia;; b Department of Nephrology, Clinical Centre of Serbia, Belgrade, Serbia;; c Primary Health Centers Rakovica, Belgrade, Serbia;; d Primary Health Centers Voždovac, Belgrade, Serbia;; e Primary Health Centers Savski Venac, Belgrade, Serbia

**Keywords:** Primary care, screening, chronic kidney disease, blood pressure, eGFR

## Abstract

**Background:** In 2009, Belgrade nephrologists and general practitioners from thirteen health centers carried out screening for chronic kidney disease (CKD). Three years later, medical records of patients from four health centers participating in the screening study were retrospectively analyzed in order to check whether general practitioners had continued to control patients at risk for CKD in accordance with the recommendations provided.

**Methods:** The study included 460 patients who visited their doctor at least once in the three-year period. Data on blood pressure, ACEI use, estimated glomerular filtration rate (eGFR) and comorbidities were taken from patients’ medical records.

**Results:** Blood pressure was not recorded in any of the three years in 42.8% and eGFR in 36.7% of the patients, but blood pressure was registered every year in 7.8% and eGFR in 4.3% of them. Over the three years, the relative number of patients with recorded blood pressure decreased from 41.7% to 17.8%, and with recorded eGFR from 41.7% to 21.5%. Multivariate linear regression found that Health Center, systolic and diastolic blood pressure and presence of hypertension were negatively associated with number of years with recorded blood pressure. Health Center, systolic blood pressure and sum of years with recorded eGFR below 60 ml/min/1.73m^2^ were associated with number of years with recorded eGFR.

**Conclusions:** Under-recording of blood pressure and eGFR in primary care health centers suggests lack of adherence to current guidelines and insufficient care of CKD patients. This implies the necessity for continuous education of physicians.

## Introduction

Chronic kidney disease (CKD) is a worldwide public health problem with increasing incidence and prevalence, poor outcome and high treatment costs. CKD affects up to 13% of the adult population [1,2]. It is estimated that about 5% of these patients will progress to end-stage renal disease (ESRD), but all CKD patients are exposed to the high risk of premature death from cardiovascular disease even in the earliest phases of CKD [3].

Early-stage CKD (stages 1–3) in most patients is asymptomatic and therefore active screening is recommended in persons at high risk for this disease [4]. As patients in the early stages of CKD are managed principally in primary health care units, doctors working there have the main role in its prevention and early detection. In addition, the most beneficial results are achieved through close cooperation of doctors in primary health care and nephrologists [5,6].

Like many nephrologists throughout the world, Belgrade nephrologists in cooperation with general practitioners from thirteen Belgrade Health Centers carried out screening for CKD in 2009. The screening included adults without previously known renal disease, but at risk for CKD, that is, patients with hypertension and/or type 2 diabetes mellitus and persons older than 60 years. The screening was preceded by educational meetings for primary care doctors on prevention and early detection of CKD, including organization of the screening study. The results of the screening were presented at a joint meeting of nephrologists and general practitioners, where nephrologists distributed prepared recommendations for prevention, screening and management of CKD. The obtained results have been published [7–9]. In order to encourage cooperation of general practitioners with nephrologists, all renal units involved in the study accepted the patients from primary care in order to guide referral and monitoring. In addition, the national guideline for prevention, diagnosis and management of CKD was introduced by the Ministry of Health of the Republic of Serbia [10].

Three years after the screening study, medical records of patients from four health centers participating in the screening study were retrospectively analyzed with the aim of checking whether general practitioners had continued to control patients at risk for CKD in accordance with the recommendations provided in the study and national guideline.

## Methods

The present analysis was conducted in four health centers where one of the authors of this paper was the coordinator-nephrologist in the original study of 2009 (VL). In these four health centers, a total of 684 patients had been examined in 2009. Out of them 195 were lost from follow-up because they changed their place of residence or left their chosen general practitioner and/or health center. In addition, 46 patients who did not visit their general practitioners once in the analyzed three-year period were excluded from the study and 31 patients died. The remaining 460 patients who visited their doctor at least once in the three-year period were included in the present study. These patients were monitored by 13 general practitioners who participated in the study voluntarily. Health Center 4 differed from others in term that only doctors working in the Diabetes counseling service of this health center participated in the 2009 screening study. Therefore, all the patients from Health Center 4 were diabetics.

After obtaining permission from the Ethics Committee of each health center, data on monitoring the patients throughout three years were taken from the electronic or paper medical records. Data on blood pressure, use of ACEI, serum creatinine levels, glomerular filtration rate estimated with MDRD4 (eGFR), comorbidities and data on hospitalizations were registered. The comorbidities included cardiovascular (ischemic heart disease, cardiac failure), obstructive pulmonary disease, gastrointestinal (ulcer, liver cirrhosis), malignancies and fractures. Also, data on the number of annual visits of each patient were taken for evaluation. Urine was analyzed in a small number of patients, but the results were not taken into further consideration.

### Statistical analysis

Continuous variables are presented as mean and standard deviation and categorical ones as frequencies. To assess differences between the examined parameters in the three examined years, the chi-square test and one-way analysis of variance with repeated measurements (ANOVA) was used depending on the variables compared. We applied univariate and multivariate linear regression to detect variables associated with number of years with recorded blood pressure or eGFR in the medical records during the three-year period. In this analysis, the following variables were included as independent ones: selected health centers denoted with serial numbers 1–4, physician (binary variable coded as 0 for physicians who included <15 patients in the present study and 1 for those who included ≥15 patients), patients’ characteristics – age, gender, number of comorbidities, systolic and diastolic blood pressure in the initial screening in 2009 and in each year of the present study, hypertension (defined as blood pressure above 140/80 mmHg) in the initial study, use of ACEI, eGFR in the initial study and in each year of the present one, stage of CKD according to KDOQI guidelines [4] in the initial study, and sum of years with recorded blood pressure >140/80 mmHg or recorded eGFR <60 mL/min/1.73m^2^ in the initial study and three years of the present study. All variables found to be significantly (*p* < .10) associated with the dependent variable were combined in multivariate linear regression analysis. Due to co-linearity between some variables several models were used in multivariate analysis.

## Results

Data on patients at the onset of the study are presented in Table 1. All patients from Health Center 4 had type 2 diabetes mellitus (DM) (explanation stated in the Methods), while in the other three centers DM was present in 5.6–15.1% of patients. Also, in comparison to the other three centers, significantly more subjects were males in Health Center 4. Average values for patients’ age, systolic and diastolic blood pressure were similar in all four health centers, where in each case the majority of patients had stage 2 CKD. The frequency of stage 1 CKD was highest in Health Center 4 and mean eGFR was significantly greater in subjects from there than in those from the other three centers (*p* < .015).

**Table 1.** Data on patients at the onset of the study.

	Health center				
	1	2	3	4	*p*
Number of patients	82	86	142	150	
Patient gender, males	29 (35.4)	29 (33.7)	48 (33.8)	76 (50.7)	.009
Patient age, years	63.2 ± 9.2	63.4 ± 8.6	62.9 ± 10.3	64.7 ± 10.7	.448
Number (%) of patients with DM	5 (6.1)	13 (15.1)	8 (5.6)	150 (100)	<.0001
Systolic BP, mmHg	140 ± 12.2	136 ± 12.9	139 ± 12.9	139 ± 14.9	.452
Diastolic BP, mmHg	82 ± 9.0	79 ± 8.6	83 ± 9.4	81 ± 8.7	.683
eGFR, ml/min/1.73m^2^	71.0 ± 13.2	72.1 ± 12.3	71.2 ± 13.0	78.6 ± 16.9	<.0001
CKD KDOQI stage					
1	7 (8.5)	16 (18.6)	12 (8.5)	43 (28.7)	
2	60 (73.2)	51(59.3)	112 (78.9)	87 (58.0)	<.0001
3	15 (18.3)	19 (22.1)	18 (12.7)	20 (13.3)	
Number of comorbidity					
0	28 (47.5)	54 (72.0)	72 (50.1)	87 (58.0)	
1	27 (45.8)	20 (26.7)	53 (37.3)	57 (38.0)	.014
≥2	4 (6.8)	1 (1.3)	17 (12.0)	6 (4.0)	

*p* – statistical significance calculated by χ^2^ test or ANOVA, as appropriate; Variable presented as number of patients (%) or mean ± standard deviation.

Comorbidities were more often present in patients from Health Centers 1 and 2, that is, for 59 (72%) patients from Health Center 1 and 75 (87%) patients from Health Center 2. The distribution of patients according to the number of comorbidities differed significantly among the four health centers. Health Center 2 had the highest number of patients with no comorbidity, while Health Center 3 had the highest proportion of patients with two or more comorbidities.

Table 2 shows the distribution of patients according to number of years with blood pressure measurements in the medical records during the three-year study depending on different variables. The mean number of years with recorded blood pressure is also given. There were no records in any year for 42.8% of the patients and only in 7.8% was blood pressure recorded in each of the three years. The percentage of patients with recorded blood pressure differed significantly between some health centers and groups of physicians. The number of years with recorded blood pressure was significantly higher in patients using ACEI and those with less co-morbidity. Analysis of the frequency of blood pressure measurements depending on different co-morbidities showed that patients with respiratory disease had blood pressure recorded most frequently. No significant differences in the number of years with recorded blood pressure were found between males and females, patients of different ages, hypertension in the initial study, different stage of CKD and the presence or absence of diabetes, but patients with blood pressure above140/80 mmHg for a higher sum of years had a significantly higher frequency of recorded blood pressure.

**Table 2.** Distribution of patients according to number of years with recorded blood pressure in the present three-year study depending on different variables examined.

	Number of years with registered blood pressure						
Variable	0*n* (%)	1*n* (%)	2*n* (%)	3*n* (%)	Mean (SD)	*p*	
Total	197 (42.8)	150 (32.6)	77 (16.7)	36 (7.8)	0.89 ± 0.95		
Health Center							
1	21 (25.6*)	39 (47.6)	13 (15.9)	9 (11.0)	1.1 ± 0.92	<.0001	
2	15 (17.4)	33 (38.4)	22 (25.6)	16 (18.6)	1.45 ± 0.99		
3	80 (56.3)	43 (30.2)	14 (9.9%)	5 (3.5)	0.61 ± 0.77		
4	81 (54.0)	35 (23.3)	28 (18.7)	6 (4.0)	0.73 ± 0.93		
Physicians							
involving ≤15 patient	83 (41.7)	61 (30.6)	34 (17.1)	21 (10.6)	0.97 ± 1.01	.041	
involving >15 patients	114 (43.7)	89 (34.1)	43 (16.5)	15 (5.7)	0.71 ± 0.87		
Patients’ characteristics							
Male	79 (43.4)	57 (31.3)	29 (15.9)	17 (9.3)		0.91 ± 0.98	.759
Female	118 (42.4)	93 (33.5)	48 (17.3)	19 (6.8)	0.88 ± 0.93		
Age in categories							
<40	6 (50.0)	4 (33.3)	2 (16.7)		0	0.67 ± 0.78	.860
40–59	49 (38.9)	44 (34.9)	24 (19.1)		9 (7.1)	0.95 ± 0.94	
60–79	138 (44.4)	98 (31.6)	48 (15.5)		26 (8.4)	0.88 ± 0.96	
≥80	4 (33.3)	4 (33.3)	3 (25.0)		1 (8.3)	1.09 ± 0.95	
Blood pressure in initial study							
≤140/80	133 (45.2)	97 (33.3)	42 (14.3)		22 (7.5)	0.84 ± 0.93	.241
>140/80	64 (38.6)	53 (31.9)	35 (21.1)	14 (8.4)		0.99 ± 0.97	
ΣBP >140/80 mmHg**							
0	30 (66.7)	8 (17.8)	5 (11.1)	2 (4.4)		0.53 ± 0.87	<.0001
1–2	146 (48.5)	124 (41.2)	18 (6.0)	13 (4.3)		0.73 ± 0.60	
3–4	0	1 (2.0)	32 (64)	17 (34.0)		2.17 ± 0.44	
Stage of chronic kidney disease (KDOQI)							
1 (eGFR >90 ml/min/1.73m^2^)	38 (48.7)	21 (26.9)	10 (12.8)	9 (11.5)		0.87 ± 1.04	.496
2 (eGFR 60–89 ml/min/1.73m^2^)	129 (41.6)	104 (33.5)	53 (17.1)	24 (7.7)		0.91 ± 0.94	
3 (eGFR 30–59 ml/min/1.73m^2^)	30 (41.7)	25 (34.7)	14 (19.4)	3 (4.2)		0.86 ± 0.88	
Diabetes						.096	
No	110 (38.7)	103 (36.3)	47 (16.5)	24 (8.5)		0.95 ± 0.94	
Yes	87 (49.4)	47 (26.7)	30 (17.0)	12 (6.8)		0.81 ± 0.95	
ACEI use							
No	135 (49.8)	69 (25.5)	45 (16.6)	22 (8.1)	0.83 ± 0.98	<.0001	
Yes	62 (32.8)	81 (42.9)	32 (16.9)	14 (7.4)		0.99 ± 0.89	
No of comorbidities							
0	106 (44.0)	79 (32.8)	42 (17.4)	14 (5.8)		0.86 ± 0.91	.031
1	70 (44.6)	47 (29.9)	27 (17.2)	13 (8.3)		0.89 ± 0. 97	
≥2	14 (50.0)	10 (35.7)	4 (14.3)	0	0.64 ± 0.98		

*p* – according to chi-square test.

* Percentage within health center or group of subjects.

** Sum of years with recorded blood pressure (BP) >140/80 mmHg in the initial and present study.

The distribution of patients according to number of years with recorded eGFR in the three-year period depending on different variables is presented in Table 3. In 36.7% of the patients, eGFR was not recorded in any year and only in 4.3% of patients was it noted every year. The frequency of recorded eGFR differed significantly between some health centers. No significant differences in the number of years with recorded eGFR were found between males and females, patients of different ages, presence of DM, or stage of CKD. On the other hand, the distribution of patients according to number of years with recorded eGFR depended significantly on ACEI treatment and number of co-morbidities. Patients with a higher sum of years with eGFR below 60 mL/min/1.73m^2^ had significantly more years with recorded eGFR.

**Table 3.** Distribution of patients according to number of years with recorded eGFR in the present three-year study depending on different variables examined.

Variable	Number of years with registered eGFR					*p*
	0 *n* (%)	1 *n* (%)	2 *n* (%)	3 *n* (%)	Mean (SD)	
Total	169 (36.7)	161 (35.0)	110 (23.9)	20 (4.3)	0.96 ± 0.88	
Health Center						
1	43 (52.4)*	35 (42.7)	4 (4.9)	0	0.52 ± 0.59	<.0001
2	20 (23.3)	26 (30.2)	32 (37.2)	8 (9.3)	1.33 ± 0.94	
3	46 (32.4)	48 (33.8)	46 (32.4%)	2 (1.4)	1.03 ± 0.84	
4	60 (40.0)	52 (34.7)	28 (18.7)	10 (6.7)	0.92 ± 0.92	
Physicians						
involving <15 patient	70 (35.5)	60 (30.5)	52 (26.4)	15 (7.6)	0.97 ± 0.87	.311
involving ≥15 patients	99 (37.6)	101 (38.4)	58 (22.1)	5 (1.9)	0.89 ± 0.81	
Patients’ characteristcs						
Male	64 (35.2)	60 (33.0)	47 (25.8)	11 (6.0)	1.03 ± 0.93	.390
Female	105 (37.8)	101 (36.3)	63 (22.7)	9 (3.2)	0.91 ± 0.85	
Age in categories						
<40	5 (41.7)	4 (33.3)	3 (25.0)	0	0.83 ± 0.83	.810
40–59	50 (40.0)	41 (32.8)	31 (24.8)	3 (2.4)	0.90 ± 0.86	
60–79	109 (35.3)	112 (36.1)	73 (23.5)	16 (5.2)	0.99 ± 0.89	
≥80	5 (45.5)	4 (36.4)	1 (9.0)	1 (9.0)	0.82 ± 1.03	
Stage of chronic kidney disease (KDOQI)						
1 (eGFR >90 ml/min/1.73m^2^)	32 (41.0)	19 (24.4)	21 (26.9)	6 (7.7)	1.01 ± 1.00	.166
2 (eGFR 60–89 ml/min/1.73m^2^)	113 (36.5)	119 (38.4)	67 (21.6)	11 (3.5)	0.92 ± 0.85	
3 (eGFR 30–59 ml/min/1.73m^2^)	24 (33.3)	23 (31.9)	22 (30.6)	3 (4.2)	1.06 ± 0.90	
ΣeGFR <60 ml/min/1.73m^2^**						
0	103 (33.6)	136 (44.3)	65 (21.2)	3 (1.0)	0.90 ± 0.86	<.0001
1–2	22 (23.6)	39 (41.9)	28 (30.1)	4 (4.3)	1.09 ± 0.85	
3–4	0	0	5 (62.5)	3 (37.5)	1.9 ± 0.58	
Diabetes						
No	104 (36.6)	98 (34.5)	74 (26.1)	8 (2.8)	0.95 ± 0.86	.140
Yes	65 (36.9)	63 (35.8)	36 (20.5)	12 (6.8)	0.97 ± 0.92	
ACEI use						
No	104 (38.4)	99 (36.5)	53 (19.6.)	15 (5.5)	0.92 ± 0.89	.040
Yes	65 (34.4)	62 (32.8)	57 (30.2)	5 (2.6)	1.01 ± 0.76	
No of comorbidities						
0	98 (40.7)	82 (34.0)	57 (23.7)	4 (1.7)	0.86 ± 0.83	.011
1	54 (34.4)	54 (34.4)	37 (23.6)	12 (7.6)	1.04 ± 0.94	
≥2	8 (28.6)	11 (39.3)	9 (32.1)	0	1.22 ± 0.79	

*p* – according to chi-square test.

* percentage within primary health center or group of subjects.

** Sum of years with registered eGFR <60 mL/min/1.73m^2^ in the initial and present study.

Trends in the percentage of patients with recorded blood pressure over three years are presented in Table 4. It can be seen that the average percentages decreased significantly over the years. Changes in relative number of patients with recorded blood pressure are shown in relation to different variables, where it is notable that the decline over time was generally similar except in the case of different health centers and ACEI usage.

**Table 4.** Percentage of patients with recorded blood pressure in medical records in three years studied.

	No	Year 1	Year 2	Year 3	*p*
Total		41.7 ± 46.4	31.3 ± 46.4	21.5 ± 41.1	<.0001
Health Center					
1	82	42.6 ± 50.0	47.6 ± 50.3	22.0 ± 41.7	<.0001
2	86	54.7 ± 50.1	65.1 ± 47.9	24.4 ± 43.2	
3	142	20.4 ± 40.5	21.1 ± 41.0	18.3 ± 38.8	
4	150	45.3 ± 50.0	14.7 ± 33.4	21.5 ± 41.1	
*p*			<.0001		
Physicians					
involving <15 patient	199	48.9 ± 50.1	30.9 ± 46.3	16.9 ± 37.6	<.0001
involving ≥15 patients	261	35.5 ± 47.9	24.8 ± 43.3	11.5 ± 32.0	
			0.292		
Patients’ characteristcs					
Male	182	41.2 ± 49.4	27.5 ± 44.8	24.2 ± 42.9	<.0001
Female	278	37.4 ± 48.5	33.8 ± 47.4	19.8 ± 39.9	
*p*			.393		
Age in categories					
<40 years	12	50.0 ± 52.2	14.4 ± 38.9	8.0 ± 28.7	<.0001
40–59 years	124	38.7 ± 48.9	37.9 ± 48.7	20.1 ± 40.9	
60–79 years	302	39.1 ± 48.9	30.5 ± 46.1	22.2 ± 41.6	
≥80 years	10	70.0 ± 48.3	30.0 ± 48.3	40.0 ± 51.6	
*p*			.278		
Blood pressure					
≤140/80 mmHg	294	35.0 ± 47.8	30.3 ± 46.0	20.8 ± 40.6	<.0001
>140/80 mm Hg	166	45.8 ± 50.0	33.1 ± 47.2	22.9 ± 42.1	
*p*			.476		
ACEI use					
Yes	189	56.0 ± 48.1	43.9 ± 50.0	19.6 ± 40.0	<.0001
No	271	40.9 ± 49.3	22.5 ± 41.8	22.9 ± 42,1	
*p*			<.0001		
eGFR					
>90 ml/min/1.73m^2^	78	32.3 ± 41.0	20.5 ± 40.6	14.4 ± 23.2	<.0001
60–89 ml/min/1.73m^2^	310	38.2 ± 48.6	33.9 ± 47.4	16.6 ± 41.2	
30–59 ml/min/1.73m^2^	72	38.9 ± 49.1	31.9 ± 47.0	18.1 ± 38.7	
*p*			.644		
No of comorbidities					
0	241	37.3 ± 48.4	27.8 ± 44.9	14.8 ± 34.2	.001
1	157	38.9 ± 48.9	22.5 ± 40.3	12.7 ± 32.3	
≥2	28	33.5 ± 47.4	20.7 ± 41.2	11.0 ± 27.7	
*p*			.330		

*p* – according to one-way ANOVA with repeated measurements.

Similar trends were observed in the relative number of patients with recorded eGFR over three years (Table 5). There were significant differences in the pattern of change of recorded eGFR between health centers and between patients using or not using ACEI. All other analyzed variables had no influence on the percentage of patients with recorded eGFR in any of the examined years.

**Table 5.** Percentage of patients with recorded eGFR in medical records in three years studied.

	No	Year 1	Year 2	Year 3	*p*
Total		41.7 ± 49.4	36.5 ± 48.2	17.8 ± 38.3	<.0001
Health Center					
1	82	27.8 ± 41.1	26.8 ± 44.6	19.7 ± 29.8	<.0001
2	86	49.8 ± 50.3	52.8 ± 48.6	21.9 ± 40.9	
3	142	48.9 ± 49.3	42.8 ± 50.1	10.0 ± 29.9	
4	150	52.7 ± 50.1	29.5 ± 49.4	22.0 ± 45.1	
*p*			<.0001		
Physicians					
involving <15 patient	199	58.4 ± 49.4	30.3 ± 46.1	19.1 ± 39.4	<.0001
involving ≥15 patients	261	50.0 ± 50.1	31.1 ± 49.4	11.4 ± 29.2	
*p*			.154		
Patients’ characteristcs					
Male	182	53.6 ± 49.9	35.7 ± 48.1	18.5 ± 41.9	<.0001
Female	278	50.2 ± 48.9	37.1 ± 48.4	14.8 ± 35.5	
*p*			.772		
Age in categories					
<40 years	12	41.7 ± 51.5	25.0 ± 45.2	6.7 ± 28.9	<.0001
40–59 years	124	36.3 ± 48.3	36.3 ± 49.3	13.7 ± 34.5	
60–79 years	302	45.4 ± 49.9	34.1 ± 48.1	20.2 ± 40.2	
≥80 years	10	30.0 ± 48.3	30.0 ± 48.3	20.0 ± 42.2	
*p*			.504		
Blood pressure					
≤140/80 mmHg	294	50.8 ± 49.2	35.8 ± 48.6	14.6 ± 35.4	<.0001
>140/80 mm Hg	166	53.3 ± 49.7	31.3 ± 47.7	18.5 ± 42.5	
*p*			.466		
ACEI use					
Yes	189	60.2 ± 49.2	30.8 ± 50.1	10.1 ± 30.2	<.0001
No	271	49.8 ± 49.6	26.6 ± 44.3	13.9 ± 42.3	
*p*			.047		
eGFR					
>90 ml/min/1.73m^2^	78	50.0 ± 50.3	28.1 ± 42.4	13.5 ± 45.9	<.0001
60–89 ml/min/1.73m^2^	310	51.4 ± 48.7	31.4 ± 48.7	15.2 ± 35.9	
30–59 ml/min/1.73m^2^	72	52.2 ± 50.3	37.1 ± 49.9	16.7 ± 37.5	
*p*			.472		
No of comorbidities					
0	241	49.8 ± 49.1	35.7 ± 48.0	11.6 ± 32.1	<.0001
1	157	44.0 ± 49.8	28.9 ± 48.9	14.0 ± 40.9	
≥2	28	42.8 ± 50.1	36.5 ± 49.0	14.7 ± 39.8	
*p*			.281		

*p* – according to one-way ANOVA with repeated measurements.

Univariate linear regression analysis was used to detect variables associated with number of years with recorded blood pressure in the medical records during the three-year period examined. Among the independent variables listed in Methods the following emerged as significantly associated with number of years with recorded blood pressure: Health center, systolic and diastolic blood pressure, the presence of hypertension defined as blood pressure above 140/80 mmHg in the initial study (negatively) and sum of years with recorded blood pressure >140/80 mmHg in the initial study and the three subsequent years and use of ACEI (positively). In addition to age and gender these variables were combined in a multivariate linear regression analysis that found the following variables to be associated significantly with number of years with recorded blood pressure in the three-year study: Health center, systolic and diastolic blood pressure and presence of hypertension (negatively) (Table 6). When the dependent variable was number of years with recorded eGFR, univariate analysis revealed that health center and systolic blood pressure were negatively, while eGFR and sum of years with recorded eGFR <60 mL/min/1.73m^2^ in the initial and present study were positively associated with the dependent variable. Multivariate analysis found health center, systolic blood pressure and sum of years with recorded eGFR <60 mL/min/1.73m^2^ to be significantly associated with number of years with recorded eGFR (Table 6).

**Table 6.** Variables associated with number of years with recorded blood pressure and eGFR in medical records in three-year period examined (multivariate linear regression).

	Dependent variables	
Independent variables	Number of years with recorded blood pressure B; beta; (95% CI); *p*	Number of years with recorded eGFR B; beta; (95% CI); *p*
Health Center	−0.20; −0.23; (−0.28 to −0.12); **<.0001**	−1.21; −1.74; (−1.64 to −0.78); **<.0001**
Patients’ characteristcs		
Gender, male	−0.03; −0.01; (−0.21 to 0.15); .764	−0.11; −0.06; (−0.28 to 0.05); .177
Age, years	0.01; 0.07; (−0.002 to 0.015); .155	0.01; 0.58; (−0.003 to 0.013); .223
Systolic BP mmHg*	−0.01; −0.20; (−0.03 to −0.003); **.017**	−0.01; −0.17 (−0.02 to 0.00); **.042**
Diastolic BP mmHg*	−0.03; −0.30; (−0.05 to −0.15); **<.0001**	
Hypertension	−1.30; −0.54; −1.47 to −1.13; **<.0001**	
ΣeGFR <60 ml/min/1.73m^2^**		0.12; 0.22; (0.06 to 0.43); **<.0001**

* BP in the initial study.

** Sum of years with recorded eGFR <60 mL/min/1.73m^2^ in the initial and present study.

## Discussion

In 2009, screening for CKD in at risk population was carried out in Belgrade by primary care physicians and nephrologists in collaboration [8]. The aim of that screening and collaboration was not only to detect persons with CKD in populations at risk but also to educate primary care physicians on how to carry out regular control of CKD markers in patients at risk for CKD. At the same time a National Guideline for Prevention, Diagnosis and Management of Chronic Kidney Disease was prepared and published by the Ministry of Health of the Republic of Serbia. However, previous investigations have shown that the guidelines were often insufficiently incorporated into practice and that many patients at risk were not being tested for CKD by primary care physicians [11–13]. The present study was undertaken in order to discover how physicians who participated in the Belgrade screening study used this experience and followed guideline recommendations. We have reviewed how often blood pressure and eGFR were recorded in the medical records of patients examined in the initial screening in 2009, and which factors influence the frequency of this recording.

Our results showed that during the three-year period following the initial study blood pressure was not recorded in any year in over 42.8% of patients and every year in less than 7.8% of them. There were significant differences between the health centers evaluated. The number of years with recorded blood pressure was significantly higher in patients using ACEI, those with less comorbidities and those from physicians who included less than 15 patients in the initial study. Patients with blood pressure above 140/80 mmHg for more years had it recorded more often. Similar results were obtained for registering eGFR in the three-year period. eGFR was not recorded in any year for 36.7% of the patients and every year in less than 4.3% of them. The frequency of eGFR records depended significantly on the health center involved, ACEI treatment and the number of co-morbidities. Patients with eGFR below 60 mL/min/1.73 m^2^ for more years had it registered more often. The percentage of patients with recorded blood pressure and eGFR declined over the three years.

It is often pointed out that primary care physicians have a key role in the early detection of CKD as well as in the use of measures for slowing down its progression. Different strategies have been attempted to influence primary care physicians to pay more attention to CKD, most often by direct education. The well-known and very extensive Kidney Early Evaluation Program (KEEP), which was initially directed to screening for CKD in subjects at risk, included education of physicians on risk factors, detection methods, complications and treatment of CKD [14,15]. In Singapore The Prevention Program initiated in 1997 included public education, screening for kidney disease and associated chronic diseases, a disease management program as well as optimization of care of patients at risk for CKD through Prevention Centers [16]. In the United Kingdom, a pay for performance (P4P) system, the Quality and Outcomes Framework, was introduced in 2004 and renal indicators were included in this system in 2006 in order to improve recognition and management of CKD in primary care by promoting the development of a CKD register. This is a unique health care system that influences primary care practice by introducing financial incentives in order to improve the outcome [17]. In Serbia, such comprehensive and general measures have not been developed, but Belgrade nephrologists have tried to improve the quality of CKD care through educational meetings and screening for CKD performed in cooperation with primary care physicians. Although during the study the collaboration of nephrologists and primary care physicians was successful and educative meetings organized before and afterwards provoked great interest among the large number of participants, the present study shows that monitoring of patients at risk for CKD is still insufficient.

Records for eGFR decreased during the three years from 41.7% of the patients to 17.8%. These results are similar to those obtained in a much larger study including 277,111 patients in the USA where at least one laboratory test was performed at the Laboratory Corporation of America during one year. Among the patients examined 19% had at least one measurement of serum creatinine, but in patients at risk for CKD (hypertension, diabetes and age >60 years) the proportion varied between 21.4% and 27.9% [11]. Ravera et al. [18] reported more favorable data, as serum creatinine was recorded in 60% of patients with hypertension monitored by general practitioners. In several practices, serum creatinine was determined in the whole patient population regardless of the presence of risk factors for CKD. The percentage of patients with recorded values varied between 17.2% and 31% [12,19,20]. In Eastern Massachusetts, an analysis of 15 health centers, where eGFR reporting was automated, showed that eGFR was evaluated in 86% of patients annually, but urine protein in only 30% of them. The rate of primary care physician recognition of CKD, defined as documentation of a CKD diagnosis on the electronic list, was 24%. This indicated that an automated eGFR reporting system is not sufficient for early detection and adequate monitoring and treatment of CKD patients [13]. In contrast to this are excellent results from the United Kingdom, where after introduction of the pay for performance system, albuminuria recordings reached 82% in registered patients in CKD stages 3–5 [21]. However, Fraser et al. [22] observed that among patients whose CKD was registered the albumin–creatinine ratio was tested in 37.0% annually, while among those not registered in 13.9%.

Hypertension is a well-known risk factor for both CKD and cardiovascular disease, which is the main cause of mortality in CKD patients. Although among the patients included in the present study 92.6% had hypertension (data not presented) blood pressure was not recorded in over 40% of them in any of the three years. Blood pressure was recorded each year in less than 10% of the patents, which is much lower than around 80% found by other authors, although the proportion of patients with hypertension was considerably lower in their studies [23–25]. Nevertheless, Filippi et al. [26] found that among 119,065 patients with a diagnosis of hypertension no blood pressure value was recorded in 27.8% during the examined year. In our initial investigation in 2009, 1466/1617 patients had hypertension [8] and 425 of them were included here. From their medical records, we learned that antihypertensive therapy was prescribed to all of them but, despite that, blood pressure was not recorded in half of them. We know from experience that many patients measure blood pressure at home and that for many older patients family members come to the doctors to get prescriptions. This indicates that hypertension is better monitored than is indicated by blood pressure data in medical records. Moreover, the values for recorded blood pressure were above 140/80 mmHg in 67.5% of patients (data not presented). This is better than in the initial study in 2009 [7] but far from satisfactory. All this indicates that there is still much room for improving the care and treatment of high blood pressure in primary health care.

In the present study, the number of patients for whom both blood pressure and eGFR were recorded, decreased by about half over the three years: that is, from 41.7% to 17.8% for eGFR and from 41.7% to 21.5% for blood pressure. In another retrospective study, eGFR was monitored annually in about 80% of patients [13,20]. Records for eGFR in our patients were not only much fewer in the first year of follow-up than in these studies but decreased further over the three years. Irregular control of the kidney function in our primary health care centers partly explains the relatively frequent late nephrology referral of patients with CKD. Although our national guideline for prevention, diagnosis and management of CKD sets out the criteria for nephrology referral in accordance with the recommendation of KDIGO guideline [6], the results obtained confirmed the well-known fact that publishing a guideline is not enough. We believe that education of primary care physicians concerning CKD detection and monitoring is of great importance for a better incorporation of the guideline in practice. The highest percentage of recorded eGFR was found in the first year after the initial screening study, and as time went on concerns about regular control of kidney function in patients at risk for CKD decreased. Although these results are not unexpected and original, we will use them in further education of primary care physicians, who should overcome their suspicion of their appropriateness and competence for the management of CKD. In addition, physicians should pay attention to the importance of educating patients about the usefulness of regular control and respect for all proposed treatment measures.

Values for proteinuria in the medical records of patients examined here were scarce, and therefore, it was not included in the analysis. In our primary health centers, measurement of albuminuria is not available and only the semiquantitative urine dipstick test is applied. In addition, at the time of this study, electronic medical records had just been introduced into primary health care centers, so most data were obtained from paper records completed personally by doctors. Also, patients received the results of laboratory analysis on paper and brought them to their doctors. It could be assumed that some serum creatinine measurements were not recorded. Now that primary health centers are computerized the laboratory results are sent electronically directly to the physicians enabling complete registration of all patient data.

Multivariate linear regression analysis indicated that health center, systolic and diastolic blood pressure and presence of hypertension were negatively associated with number of years with recorded blood pressure in the three-year study, while health center, systolic blood pressure and sum of years with recorded eGFR <60 mL/min/1.73 m^2^ were associated with number of years with recorded eGFR. Thus, recording of both blood pressure and eGFR depended significantly on the health center involved. Comparison of patients’ data at the outset of the present study showed significant differences in mean eGFR and number of comorbidities between the four health centers but the differences could hardly be connected with blood pressure and eGFR recording. It has already been mentioned that all doctors from Health Center 4 worked in Diabetes counseling service and therefore all the patients from Health Center 4 were diabetics. However, although these patients were controlled in a specialized diabetes counseling, blood pressure and eGFR recording was no better than in other centers. Decline in relative number of patients with recorded blood pressure and eGFR over three years was similar in Health Center 4 to some other centers. Even more, in the second year of the study, percentage of patients with recorded blood pressure and eGFR in Health Center 4 was lower than in the other two centers. All this shows that although it is considered that both physicians and patients with DM know the importance of blood pressure as well as kidney function control, it is not implemented in practice.

Brady and O’Donoghue [27] also described wide variation in documented CKD prevalence between practices, which could not be explained by practice characteristics or demographic data. Crinson et al. [28] described very large differences in practitioners’ views of CKD and embracing of guidance. It could be assumed that such diversity exists among our doctors and that this led to disparity in recording blood pressure and eGFR between the health centers.

In health services where blood pressure and eGFR were determined in the whole population of patients, records of blood pressure were more frequent in those with risk factors for cardiovascular disease [26], while records of kidney function were more frequent in high-risk patients (older age, diabetes, hypertension, lower eGFR) [11–13,20,22] than in individuals without these risk factors. This indicates that these physicians paid more attention to high-risk patients. Our results suggest something else. Patients whose blood pressure and eGFR were recorded less often had higher blood pressure and lower eGFR, indicating that irregular checkups with the physician were related to poorer control of blood pressure and worse renal function.

Our study has some limitations. First, the present study included data from four out of thirteen health centers included in the initial screening study in 2009. Therefore, the results obtained cannot be generalized in relation to the initial study, and certainly not in relation to all primary health centers. Although it cannot be excluded that data from a larger number of centers might show a different picture, results obtained here shed some light on the quality of care of patients at risk for CKD and these are the first such data from our primary health care. Secondly, data on recording blood pressure and eGFR might be underestimated due to analysis of data personally recorded by physicians. It is possible that blood pressure and serum creatinine were measured but not recorded. On the other hand, the use of routine data from medical records of primary health centers provides a realistic view on how blood pressure and eGFR are recorded in our primary health care. The data obtained represent a starting point for further research, as well as a direction for further action in terms of improving the care of patients at risk for CKD.

Under-recording of blood pressure, as one of the main risk factors for CKD, as well as under-recording of eGFR, as a marker of CKD, suggests lack of adherence to current guidelines and therefore insufficient care of CKD patients. Continuous education of primary care physicians is an essential step towards improved prevention, early detection and management of CKD.

## Ethical approval

Ethics Committee of each health center involved in the study has given consent to research.
